# Effects of omeprazole or pantoprazole on platelet function in non-ST-segment elevation acute coronary syndrome patients receiving clopidogrel

**DOI:** 10.1186/s40779-016-0107-0

**Published:** 2016-12-15

**Authors:** Ruo-Xi Gu, Xiao-Zeng Wang, Jing Li, Jie Deng, Xing-Xing Li, Jiao Wang

**Affiliations:** Department of Cardiology, General Hospital of Shenyang Military Region, Shenyang, 110840 China

**Keywords:** Omeprazole, Pantoprazole, Clopidogrel, Platelet response, Non-ST-segment elevation acute coronary syndrome

## Abstract

**Background:**

This study evaluated the effect of omeprazole or pantoprazole on platelet reactivity in non-ST-segment elevation acute coronary syndrome (NSTE-ACS) patients receiving clopidogrel.

**Methods:**

Consecutive patients with NSTE-ACS (*n* = 620) from general hospital of Shenyang Military Command were randomized to the omeprazole or pantoprazole (20 mg/d) group (1:1), and received routine dual antiplatelet treatment. Patients’ reversion rate of adenosine diphosphate-induced platelet aggregation (ADP-PA) was assessed at baseline, 12 to 24 h after administration of medication, and after 72 h of percutaneous coronary intervention (PCI). The primary endpoint of the study was platelet reactivity assessed with ADP-PA at 30 days after PCI. Adverse events (AEs) were recorded for 30-day and 180-day follow-up periods.

**Results:**

There were no significant differences between both the groups in platelet response to clopidogrel at 12–24 h after drug administration (54.09% ± 18.90% *vs* 51.62% ± 19.85%, *P* = 0.12), 72 h after PCI (52.15% ± 19.45% *vs* 49.66% ± 20.05%, *P* = 0.18), and 30 days after PCI (50.44% ± 14.54% *vs* 48.52% ± 15.08%, *P* = 0.17). The rate of AEs did not differ significantly between groups during the 30-day (15.2% *vs* 14.8%, *P* = 0.91) and 180-day (16.5% *vs* 14.5%, *P* = 0.50) follow-up periods after PCI.

**Conclusions:**

The addition of omeprazole or pantoprazole to clopidogrel did not restrict the effect of platelet aggregation by reducing the conversion of clopidogrel. Compared with clopidogrel alone, pantoprazole-clopidogrel and omeprazole-clopidogrel combinations did not increase the incidence of adverse clinical events during 30-day and 180-day follow-up periods after PCI.

**Trial registration:**

The study is registered in the National Institutes of Health website with identifier NCT01735227. Registered 14 November 2012.

## Background

Numerous clinical trials and physician practices have shown dual antiplatelet therapy (DAPT) to play a vital role in the treatment of patients with acute coronary syndrome (ACS) undergoing percutaneous coronary implantation (PCI) [1]. Although DAPT can effectively inhibit coronary stent thrombosis in patients with ischemic heart disease, reduce the incidence of major adverse cardiovascular events (MACE), and control the rate of readmission, thus greatly improving patients’ quality of life, it can also increase the risk of gastrointestinal complications. Therefore, in 2010, the American College of Cardiology Foundation, the American College of Gastroenterology, and the American Heart Association recommended that proton pump inhibitors (PPIs) be prescribed to patients undergoing DAPT to decrease the risk of gastrointestinal bleeding [2–5]. However, medications metabolized by cytochrome P450 (CYP450), such as PPIs, have been shown to decrease the effectiveness of clopidogrel, and observational studies have suggested that PPIs might restrict the effect of platelet aggregation by reducing the conversion of clopidogrel into its active form through competitive inhibition of the CYP2C19 isoenzyme, based on pharmacodynamic data. In addition, several reports have shown that concomitant use of clopidogrel and PPIs is associated with an increased risk of adverse outcomes compared with the use of clopidogrel alone [6–10]. However, Dunn et al. [11] showed no significant difference in the number of adverse events (AEs) following PCI in patients with and without PPI. While omeprazole has been associated with reduced clopidogrel efficacy, as assessed by the platelet reactivity index vasoactive stimulated phosphoprotein, new PPIs such as pantoprazole and esomeprazole have been shown to have no effect on biological response to clopidogrel [12]. Because many PPIs are metabolized to varying degrees by CYP2C19, the reported negative omeprazole-clopidogrel drug interaction may not be caused by a class effect [13, 14].

PPIs are usually prescribed to patients undergoing dual antiplatelet therapy to decrease the risk of gastrointestinal bleeding. However, there has been an increased incidence of MACE because of the interaction of PPIs and clopidogrel. Therefore, this study was conducted to evaluate the effect of omeprazole or pantoprazole on platelet reactivity in non-ST-segment elevation acute coronary syndrome (NSTE-ACS) patients receiving clopidogrel.

## Methods

### Study design and population

**Fig. 1:**
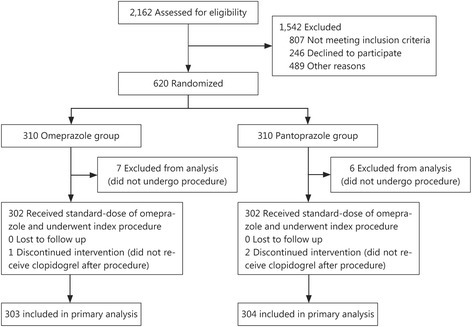
Patient flowchart

This prospective, randomized controlled clinical trial (NCT01735227) was conducted at department of cardiology, General Hospital of Shenyang Military Region to compare the influence of omeprazole or pantoprazole on the antiplatelet effect of clopidogrel in patients undergoing PCI for NSTE-ACS. In our study, we defined NSTE-ACS as unstable angina (UA) and NSTEMI because all STEMI patients received immediate emergency PCI if the clinical symptoms were compatible with acute myocardial ischemia within 12 h before admission. Patients aged 18 to 75 years were eligible to be enrolled if they had coronary artery disease and opted to undergo PCI. Eligible patients were randomly assigned to 2 groups that received either omeprazole or pantoprazole (in a 1:1 ratio; random envelope provided by the CRO company). The major exclusion criteria included the use of glycoprotein IIb/IIIa inhibitors within 24 h before enrollment or use of cilostazol within 7 days before enrollment, contraindication to DAPT, and a history of severe systemic bleeding or greatly increased risk of bleeding. Patients were also excluded if they had New York Heart Association grade III or IV cardiac function, PCI or coronary artery bypass grafting (CABG) within the past year, persistent atrial fibrillation requiring long-term warfarin, or prior use of a PPI or clopidogrel. Patients with serious liver or kidney dysfunction were also excluded. The design of the study is depicted in Fig. 1.

For 80% power to detect the noninferiority of omeprazole-clopidogrel or pantoprazole-clopidogrel to clopidogrel alone, a sample size of 295 patients were assigned to each of the omeprazole and pantoprazole groups in a 1:1 ratio. Patients were required to achieve a two-sided significance level of 0.05. Taking into account an attrition rate of about 5%–10%, at least 620 patients were enrolled in our study.

### Study protocol

The study was approved by the ethics committee of General Hospital of Shenyang Military Region. The study was performed in accordance with ethical principles that have their origin in the Declaration of Helsinki and are consistent with ICH/Good Clinical Practice. All patients provided written informed consent before enrollment. All patients received a loading dose of 300 mg each of clopidogrel and aspirin, and were randomized to take 20 mg daily of either omeprazole (Losec MUPS; AstraZeneca, London, UK) or pantoprazole (Tecta; Takeda Pharmaceuticals International GmbH, Konstanz, Germany) and received routine dual antiplatelet treatment (aspirin 300 mg and clopidogrel 75 mg daily). PPI therapy was resumed in both groups following completion of the primary endpoint of the study.

Blood samples (two 3.6 ml tubes) were taken to measure the reversion rate of ADP-PA, which was defined as 20 μmol/L adenosine diphosphate-induced platelet aggregation by light transmittance aggregometry (LTA) before randomization, 12 to 24 h after drug administration, and 72 h after PCI. The upper, platelet-rich plasma layer was prepared by centrifuging blood samples for 12 min; the remaining platelet-poor plasma was obtained by centrifuging for 15 min for use as a blank control. Platelet aggregation rate was determined with an AggRAM® eight-channel Advanced Modular System for Platelet Aggregation Ristocetin Cofactor (Helena Laboratories, Beaumont, TX, USA) by a skilled technician. An assessment of platelet reactivity by LTA was scheduled for all enrolled patients 30 days after PCI. Results were used in the primary-endpoint evaluation.

### Study endpoints

The primary endpoint of the study was platelet reactivity of 20 μmol/L ADP-PA by LTA 30 days after PCI. The secondary endpoints were clinical events during the 30-day and 180-day follow-up periods after PCI, which included stent thrombosis, MACE (cardiac death, non-fatal myocardial infarction, or ischemic symptom-driven target-vessel revascularization [TVR] or non-TVR), all-cause death, thrombolysis in myocardial infarction (TIMI) bleeding events, stroke, and adverse drug reactions. Stent thrombosis was defined according to the Academic Research Consortium criteria, and only definite stent thrombosis was counted [15]. Myocardial infarction was defined as a creatine kinase myocardial band level that was more than twice the upper limit of normal range and either symptoms consistent with acute myocardial infarction or electrocardiographic changes in at least two contiguous leads (pathologic Q waves 0.04 s in duration or persistent ST-segment elevation or ST-segment depression > 0.1 mV). TVR included intervention because of recurrence in any part of the original vessel. Bleeding events were divided into major or minor bleeding on the basis of thrombolysis in TIMI bleeding classification as follows: major bleeding = intracranial hemorrhage or a >5 g/dl decrease in hemoglobin concentration or a >15% absolute decrease in hematocrit; moderate bleeding = observed blood loss (a > 4 g/dl decrease in hemoglobin concentration or a > 12% decrease in hematocrit); minor bleeding = no observed blood loss (a > 3 g/dl decrease in hemoglobin concentration or a > 10% decrease in hematocrit) [16]. Stroke was defined as persistent loss of neurological function developing after primary PCI and an acute lesion identified with magnetic resonance imaging. Adverse drug reactions in our study included reactions to aspirin, clopidogrel, nitrates, and statins.

All patients had a follow-up evaluation at a clinic visit or *via* telephone contact 30 and 180 days following PCI. The China Cardiovascular Research Foundation (CCRF), an independent clinical research organization, was responsible for database management, safety monitoring, and evaluation of AEs. All AEs were adjudicated by a blinded, independent clinical-events committee. The CCRF reviewed the data periodically to identify any potential safety issues.

### Statistical analysis

This study used intention-to-treat analysis data sets for statistical analysis. Comparisons among normally distributed continuous variables are presented as mean ± standard deviation and were compared using Student’s unpaired *t-*test. Categorical variables are expressed as frequencies and percentages. Comparisons between groups were made using the Chi-square or Fisher exact test for categorical variables and nonparametric statistical testing (Mann-Whitney *U*-test) for continuous variables. Values of *P <* 0.05 were considered statistically significant. All analyses were performed with the IBM SPSS Statistics for Windows, Version 21.0 (IBM Corp, Armonk, NY, USA).

## Results

**Fig. 2:**
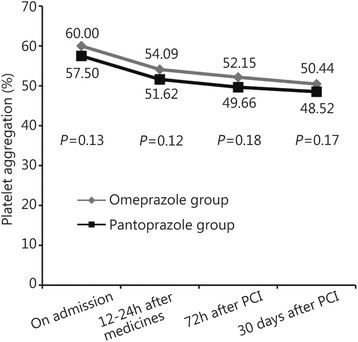
Changes in ADP-PA between the two groups of patients

From October 2012 to September 2013, a total of 620 patients with NSTE-ACS were enrolled from general hospital of Shenyang military region. Thirteen patients did not undergo angiography and were excluded. The remaining 607 patients were included, with 303 patients allocated to the omeprazole group and 304 to the pantoprazole group. All patients completed 30 and 180 days follow-up periods after PCI. All groups were generally well balanced with regard to baseline demographic, clinical, and procedural characteristics. Baseline and procedural characteristics of the study patients are shown in Tables 1 and 2. Of those 607 patients, 602 (99.8%) underwent coronary angiography after an oral loading dose of aspirin 300 mg and clopidogrel 300 mg on admission. Successful PCI, which was defined as recovery of coronary flow to TIMI grade 2 to 3 and residual stenosis <50%, was achieved in all patients. Two patients died during hospitalization: one from stent thrombosis and the other because of traumatic brain injury. There were no differences between the omeprazole and pantoprazole groups in platelet response to clopidogrel on admission, 12 to 24 h after drug administration, and 72 h and 30 days after PCI (Fig. 2).

**Table 1:** Baseline patient characteristics

Characteristic	Omeprazole group (*n* = 303)	Pantoprazole group (*n* = 304)	*P* value
Age (year)	59.15 ± 8.75	58.76 ± 8.50	0.58
Male [*n* (%)]	209 (69.0)	215 (70.7)	0.66
Cardiovascular risk factors			
BMI (kg/m^2^)	25.57 ± 3.86	25.54 ± 3.43	0.92
Hypertension [*n* (%)]	199 (65.7)	186 (61.2)	0.27
Dyslipidemia [*n* (%)]	135 (44.6)	125 (41.1)	0.41
Diabetes mellitus [*n* (%)]	84 (27.7)	82 (27.0)	0.86
Active smoking [*n* (%)]	170 (56.1)	171 (56.3)	1.00
Previous myocardial infarction [*n* (%)]	48 (15.8)	46 (15.1)	0.82
Previous transient ischemic attack/stroke [*n* (%)]	27 (8.9)	27 (8.9)	1.00
Final clinical diagnosis [*n* (%)]			
UA	262 (84.3)	264 (86.8)	0.90
NSTEMI	42 (13.9)	39 (12.8)	0.72
Laboratory parameters			
WBC (×10^12^/L)	7.01 ± 1.85	6.91 ± 1.74	0.48
RBC (×10^9^/L)	6.04 ± 0.34	4.52 ± 0.55	0.32
PLT (×10^12^/L)	211.16 ± 51.24	210.42 ± 51.89	0.86
Hb (g/L)	140.66 ± 15.19	141.24 ± 13.87	0.67
Creatinine (mg/dl)	71.73 ± 23.13	69.77 ± 22.95	0.27
CRP (mmol/L)	3.73 ± 6.47	4.82 ± 17.54	0.59
TNT (ng/ml)	0.08 ± 0.32	0.07 ± 0.31	0.62
Blood glucose (mmol/L)	6.40 ± 2.46	6.75 ± 3.39	0.21
Total cholesterol (mmol/L)	3.88 ± 1.05	3.86 ± 0.99	0.82
Total triglycerides (mmol/L)	1.97 ± 1.32	1.92 ± 1.42	0.95
Low-density lipoprotein cholesterol (mmol/L)	2.18 ± 0.95	2.14 ± 0.87	0.56
High-density lipoprotein cholesterol (mmol/L)	1.09 ± 0.72	1.11 ± 0.80	0.80
Baseline ADP-PA (%)	60.00 ± 20.87	57.50 ± 19.73	0.13

*BMI* Body mass index, *UA* Unstable angina, *NSTEMI* Non-ST segment elevation myocardial infarction, *WBC* White blood cell, *RBC* Red blood cell, *PLT* Platelet, *Hb* Hemoglobin, *CRP* C-reactive protein, *ADP-PA* Adenosine diphosphate-induced platelet aggregation

**Table 2:** Procedural characteristic during hospitalization

Characteristic	Omeprazole group (*n* = 303)	Pantoprazole group (*n* = 304)	*P* value
Systolic blood pressure (mmHg)	140.01 ± 22.16	138.86 ± 18.36	0.55
Diastolic blood pressure (mmHg)	79.81 ± 13.88	80.05 ± 11.11	0.84
Left ventricular ejection fraction (%)	62.5 ± 11.2	63.7 ± 14.7	0.30
Medications at discharge [*n* (%)]			
Statin	297 (98.0)	299 (98.4)	0.77
ACE inhibitors	160 (53.0)	143 (47.0)	0.17
Angiotensin receptor blocker	86 (28.4)	93 (30.7)	0.59
Beta-blockers	226 (74.6)	244 (80.3)	0.10
Calcium-channel blockers	77 (25.4)	79 (26.0)	0.93
Nitrate	274 (90.4)	268 (88.2)	0.43
Diuretics	35 (11.6)	33 (10.9)	0.80
Glycoprotein IIb/IIIa antagonists	32 (14.0)	22 (9.5)	0.15

**Fig. 3:**
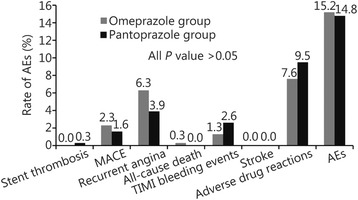
The rate of adverse clinical events in the two groups during 30-day follow-up

There were no significant differences between the two groups in coronary angiography clinical data (Table 3), and the 30-day and 180-day follow-up rates were 100%. During the 30-day follow-up period, there was one case of stent thrombosis in the pantoprazole group and none in the omeprazole group, but the difference was not significant. There were no significant between-group differences in rates of MACE. No major or minor bleeding occurred in either group, and the rates of minimal bleeding, which occurred in both groups, did not differ significantly between the groups. Although one patient died from traumatic brain injury in the omeprazole group, there was no significant difference between the groups in all-cause death. No patient in either group experienced stroke. There was no significant difference in the rate of adverse drug reactions between the omeprazole and pantoprazole groups. AEs for the two groups during the 30-day follow-up period are shown in Table 4 and Fig. 3.

**Table 3:** Analysis of coronary angiography

Characteristic	Omeprazole group (*n* = 303)	Pantoprazole group (*n* = 304)	*P* value
Target coronary vessel [*n* (%)]			
Single-vessel	84 (29.2)	94 (32.3)	0.42
Multivessel	206 (71.5)	197 (67.7)	0.32
Localization of culprit lesion [*n* (%)]			
Left main coronary artery	40 (13.2)	38 (12.5)	0.81
Left anterior descending artery	219 (72.3)	225 (74.0)	0.65
Left circumflex artery	142 (46.9)	135 (44.4)	0.57
Right coronary artery	164 (54.1)	158 (52.0)	0.63
Other artery	113 (37.3)	91 (29.9)	0.06
Baseline blood flow in the culprit vessel [*n* (%)]			
TIMI 0	47 (15.7)	35 (11.6)	0.16
TIMI 1	18 (6.0)	17 (5.6)	0.86
TIMI 2	33 (11.0)	27 (8.9)	0.42
TIMI 3	202 (67.3)	223 (73.8)	0.09
Final blood flow in the culprit vessel [*n* (%)]			
TIMI 0	8 (2.7)	11 (3.6)	0.64
TIMI 3	281 (93.7)	285 (94.4)	0.73
Characteristics of coronary angiography			0.23
Number of implanted stents (*n*)	1.29 ± 1.63	1.18 ± 1.07	0.23
Average stent diameter (mm)	2.24 ± 1.42	2.22 ± 1.44	0.81
Average length of implanted stents (mm)	18.62 ± 12.50	18.55 ± 12.98	0.94
Contrast media [*n* (%)]			
Isotonic	25 (8.4)	27 (9.0)	0.85
Non-isotonic	274 (91.6)	273 (91.0)	0.85
Median contrast agent dose (ml)	220.20 ± 134.97	205.73 ± 118.83	0.16

**Table 4:** AEs during 30-day follow-up

Characteristic	Omeprazole group (*n* = 303)	Pantoprazole group (*n* = 304)	*P* value
Stent thrombosis [*n* (%)]	0	1 (0.3)	1.00
MACEs [*n* (%)]	7 (2.3)	5 (1.6)	0.58
Cardiac death	1 (0.3)	0	0.50
Myocardial infarction	0	0	—
Ischemic symptoms driven target vessel revascularization	1 (0.3)	0	0.50
Non-target vessel revascularization	5 (1.7)	5 (1.6)	1.00
Recurrent angina [*n* (%)]	19 (6.3)	12 (3.9)	0.20
All-cause death [*n* (%)]	1 (0.3)^a^	0	0.50
TIMI bleeding events [*n* (%)]			
Major	0	0	—
Moderate	0	0	—
Minor	4 (1.3)	8 (2.6)	0.38
Stroke [*n* (%)]	0	0	—
Adverse drug reactions [*n* (%)]	23 (7.6)	29 (9.5)	0.47
AEs [*n* (%)]	46 (15.2)	45 (14.8)	0.91

^a^One patient died due to brain injury in the omeprazole group

**Fig. 4:**
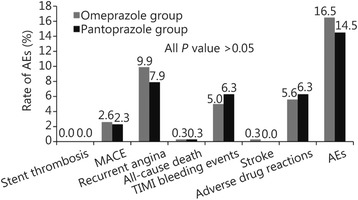
The rate of AEs in both the groups during 180-day follow-up

During the 180-day follow-up period, there was no stent thrombosis in either group. One major and three minor bleeding events occurred in the pantoprazole group, but there was no significant difference in the rate of minimal bleeding between the groups. There was no significant association of either intervention with all-cause death. One patient in the omeprazole group died of lung cancer and one patient in the pantoprazole group died of acute brainstem hemorrhage (Table 5 and Fig. 4).

**Table 5:** AEs during 180-day follow-up

Characteristic	Omeprazole group (*n* = 303)	Pantoprazole group (*n* = 304)	*P* value
Stent thrombosis [*n* (%)]	0	0	—
MACEs [*n* (%)]	8 (2.6)	7 (2.3)	0.80
Cardiac death	1 (0.3)	0	0.50
Myocardial infarction	0	0	—
Ischemic symptoms driven target vessel revascularization	3 (1.0)	1 (0.3)	0.37
Non-target vessel revascularization	6 (2.0)	6 (2.0)	1.00
Recurrent angina [*n* (%)]	30 (9.9)	24 (7.9)	0.40
All-cause death [*n* (%)]	1 (0.3)^a^	1 (0.3)^b^	1.00
TIMI bleeding evens [*n* (%)]	15 (5.0)	19 (6.3)	0.60
Major	0	1 (0.3)	1.00
Moderate	0	3 (1.0)	0.25
Minor	16 (5.3)	14 (4.6)	0.71
Stroke [*n* (%)]	1 (0.3)	0	0.50
Adverse drug reactions [*n* (%)]	17 (5.6)	19 (6.3)	0.86
AEs [*n* (%)]	50 (16.5)	44 (14.5)	0.50

^a^One patient died due to lung cancer in the omeprazole group

^b^One patient died due to acute brainstem hemorrhage in the pantoprazole group

## Discussion

Clopidogrel combined with aspirin in patients undergoing PCI has been recommended because of its ability to reduce cardiovascular events. It is well known that gastrointestinal hemorrhage is the most common serious bleeding complication of antiplatelet therapy, especially in ACS patients. Therefore, PPIs are often prescribed to prevent gastrointestinal tract bleeding during DAPT [17]. Recent studies, however, have suggested that PPIs might reduce the antiplatelet effect of clopidogrel through inhibition of hepatic CYP2C19 [18–20]. Clopidogrel is a prodrug that requires hepatic CYP450-dependent biotransformation into an active metabolite, which irreversibly blocks the P2Y12 ADP receptor [21, 22]. The genotype of this enzyme has been divided into three groups: rapid extensive metabolizers, intermediate metabolizers, and poor metabolizers. There are genetically interethnic differences in the frequencies of poor metabolizers of CYP2C19: 2.5% in white Americans, 2.0% in African Americans, 3.5% in white Europeans, and 19.8% in the Chinese-Han population. Because of the much greater prevalence of CYP2C19 loss-of-function alleles in Asians than in other populations, the influence of drug interaction might be more apparent in Asian people [23]. A nationwide population study to investigate the influences of concomitant use of clopidogrel and PPIs would be necessary to elucidate CYP2C19 polymorphisms. A Taiwanese population-based study has reported that the concomitant use of clopidogrel and PPIs is associated with an increased risk of rehospitalization and mortality in patients undergoing PCI. Thus, prior studies have suggested that an attenuation of the antiplatelet effect of clopidogrel by PPIs could lead to adverse clinical outcomes by decreasing the efficacy of clopidogrel. Previous reports have demonstrated that concomitant use of PPI and clopidogrel after PCI was associated with an increased risk of rehospitalization and mortality, and higher rates of cardiovascular events and increased risk of adverse outcomes have been shown in patients undergoing treatment with PPI-clopidogrel combination compared with clopidogrel alone [9].

In contrast, Ray et al. reported that concomitant use of clopidogrel with a PPI did not increase the incidence of serious cardiovascular disease but was associated with a 50% reduction in the incidence of hospitalizations for gastrointestinal bleeding. Several other studies have also reported that PPI use is not associated with an increased risk of cardiovascular events or mortality in patients taking clopidogrel [3–10, 12].

Kwok et al. [24] reviewed 23 studies involving 93,278 patients, and their meta-analysis of 13 of these studies showed no significant association between PPI use and overall mortality. In the latest study, the COGENT trial, 3761 patients with an ACS were randomized at the time of PCI to take clopidogrel and omeprazole or clopidogrel alone, and no evidence of any adverse clinical interaction was observed [3]. The results showed that the majority of patients who were discharged on a PPI were prescribed lansoprazole (77.7%), followed by omeprazole (17.8%) and rabeprazole (4.5%). Kenngott et al. [25] indicated that the clopidogrel-PPI interaction does not seem to be a PPI class effect, and that rabeprazole did not interfere with the clopidogrel effect in a subject with a clear omeprazole-clopidogrel interaction. Other studies have suggested that the omeprazole-clopidogrel interaction was stronger than that of clopidogrel with other PPIs [3, 6]. However, in vivo data show that lansoprazole has the potential to have the same or greater potency than omeprazole. In addition to clinical factors such as drug interactions, genetic factors, including polymorphisms of the CYP system, are important [14, 17]. The CYP2C19 polymorphism in particular appears to play a key role in both clopidogrel and PPI metabolisms. It could be that reduction in the functional alleles of CYP2C19 is associated with lower levels of the active metabolite of clopidogrel, greater levels of platelet reactivity, and a greater rate of adverse clinical outcomes in patients receiving clopidogrel. The COGENT trial is the only available controlled randomized study addressing the clinical relationship between PPIs and clopidogrel, and no evidence of any adverse clinical interaction was reported. However, 94% of the population in the COGENT trial was Caucasian, and the expected prevalence of CYP2C19 loss-of-function alleles was 2–3% [3].

The relative risk of gastrointestinal bleeding in patients receiving DAPT and the effectiveness of PPIs in preventing such bleeding are obviously critical to any decision to initiate concomitant therapy with a PPI and clopidogrel. Evidence has shown that PPIs are effective in preventing gastrointestinal bleeding in patients receiving antiplatelet therapy, including aspirin or clopidogrel, but the clinical outcomes of various studies have thus far shown conflicting results [3–6].

Therefore, coronary heart disease patients who receive clopidogrel therapy can have different individual responses. We used ADP 20 μmol/L as an inducer and evaluated platelet function by LTA. LTA is the classic method for testing platelet function. In 2010, the POPular study by Breet et al. [26], compared the correlations between incidence of clinical events and six methods of platelet function measurement: Verify*Now®*, LTA, Plateletworks™, DiaMed Impact-R, PFA-100®, and Innovance® PFA P2Y. Their results showed that Verify*Now®* (13.3% *vs* 5.7%, *P* < 0.001), LTA (11.7% *vs* 6.0%, *P* < 0.001), and Plateletworks® (12.5% *vs* 6.1%, *P* = 0.005) were better correlated with clinical results than the other methods. However, there is still no stronger predictive value than area under the curve (0.61–0.63).

The platelet function test results for individual DAPT is currently a research focus, possibly because on-treatment platelet reactivity is not a modifiable risk factor for thrombotic events after PCI. On the other hand, thrombosis is not only associated with antiplatelet therapy, but also influenced by many environmental and genetic factors, such as atherosclerotic plaque instability; heart, kidney, or other vital organ dysfunction; or sluggish blood flow.

Although in vitro studies have suggested a possible effect and several retrospective analyses were supportive of an adverse clinical outcomes, recent data from prospective controlled trials do not support an adverse cardiovascular outcome, nor do they show a clear relationship between PPI use and adverse outcomes. Therefore, this topic remains controversial. Careful risk-benefit assessment is required before prescribing PPIs to individual patients receiving DAPT, as indicated in the statement by the US Food and Drug Administration.

Safety is a concern in antiplatelet therapy, but in the present study, there were no significant differences between the groups in the rates of muscle pain, liver function test results, gastrointestinal disorders, or incidence of edema or rash during the 30-day and 180-day follow-up periods.

Our trial had several limitations. Firstly, a major limitation of the study was that CYP2C19 polymorphisms were not considered; therefore, a future study to investigate the influences of concomitant use of clopidogrel with different PPIs in different ethnicities would be necessary. Secondly, the follow-up period was short and the sample relatively small, and so a longer follow-up period and larger sample size will be necessary to explore the effect of PPIs on clopidogrel. Thirdly, we used LTA to test platelet aggregation in our study because of storage time, beside detection just as Verify*Now®* may reduce detection error. Finally, as with any prospective study, a causal relation cannot be confirmed, nor can confounding by unknown or unmeasured factors be entirely excluded. Because this was not a blinded study, there was a potential for observational bias.

## Conclusion

In conclusion, the study results suggest that combination therapy with omeprazole or pantoprazole and clopidogrel does not restrict ADP-induced platelet aggregation. There was no apparent association between clinical events and clopidogrel-omeprazole or clopidogrel-pantoprazole combination in NSTE-ACS patients. The clinical impact of this strategy needs to be confirmed by long-term follow-up outcome studies.

## Abbreviations

ACS: Acute coronary syndrome
ADP-PA: Adenosine diphosphate-induced platelet aggregation
AEs: Adverse events
CABG: Coronary artery bypass grafting
CYP450: Cytochrome P450
DATP: Dual antiplatelet therapy
LTA: Light transmittance aggregometry
MACE: Major adverse cardiovascular events
NSTE-ACS: Non-ST-segment elevation acute coronary syndrome
PCI: Percutaneous coronary intervention
PPIs: Proton pump inhibitors
TIMI: Thrombolysis in myocardial infarction
TVR: Target-vessel revascularization
UA: Unstable angina
